# Extensive Arterial and Venous Thrombosis in a Female With a Known Untreated Polycystic Ovarian Syndrome: A Case Report

**DOI:** 10.7759/cureus.34251

**Published:** 2023-01-26

**Authors:** Salim A Al Busaidi, Muzna Al-Farsi, Juhaina S Al-Maqbali, Masoud Salim Kashoob, Hatem Farhan, Bader Al Rawahi, Abdullah M Al Alawi

**Affiliations:** 1 Medicine, Sultan Qaboos University Hospital, Muscat, OMN; 2 Family Medicine, Oman Medical Specialty Board, Muscat, OMN; 3 Clinical Pharmacy, Sultan Qaboos University Hospital, Muscat, OMN; 4 Internal Medicine, Oman Medical Specialty Board, Muscat, OMN; 5 Hematology, Sultan Qaboos University Hospital, Muscat, OMN

**Keywords:** atrial septal defect, case report, pulmonary embolism, deep venous thrombosis, polycystic ovarian syndrome

## Abstract

Polycystic ovarian syndrome (PCOS) is a common heterogeneous endocrine disease associated with a twofold higher risk of stroke and venous thromboembolism (VTE). An 18-year-old female presented to the emergency department (ED) with a one-hour history of right-side body weakness, facial asymmetry, and altered mental status. The patient had poor mentation and was unable to protect her airway. She was intubated and admitted to the intensive care unit (ICU). She was diagnosed with polycystic ovarian syndrome three years ago; however, she was not on active treatment at the time of presentation. She received two doses of the BNT162b2 mRNA COVID-19 vaccine, and her last dose was six months before the current presentation. A workup showed that she had extensive arterial and venous thrombosis. Later during investigations, she was found to have a complex atrial septal defect (ASD) with a left-to-right shunt. This case reports a management approach for a young female with untreated polycystic ovarian syndrome that predisposed her to develop deep vein thrombosis (DVT), pulmonary embolism (PE), and ischemic stroke due to atrial septal defect with possible transient shunt reversal.

## Introduction

Venous and arterial thromboses have been historically recognized as diseases of different etiologies. However, more recent evidence has demonstrated an association between arterial and venous thromboses, probably due to the presence of a common risk factor [1,2]. The shared risk factors include age, smoking, metabolic syndrome, cancers, elevated estrogen levels during pregnancy, combined oral contraceptive pills, immobility, and thrombophilias [1,3].

Polycystic ovarian syndrome (PCOS) is a common heterogeneous endocrine disease associated with multiple complications, including diabetes mellitus, hypertension, dyslipidemia, obesity, and metabolic syndrome [4], which are significantly associated with increased cardiovascular diseases, ischemic stroke, and venous thromboembolism (VTE) [5-7].

Unusual thrombotic events have been recently reported in patients shortly after receiving COVID-19 vaccinations [8]. These thrombotic events included extensive development of deep vein thrombosis (DVT), pulmonary embolism (PE), cerebral venous sinus thrombosis, central nervous system thrombosis, and stroke [8-15].

Here, we report the case of an 18-year-old female with PCOS who was on progestin-only pills and received two doses of BNT162b2 mRNA COVID-19 (Pfizer-BioNTech) vaccine six months before presentation. She was admitted with multiple thromboses, including acute malignant left middle cerebral artery (MCA) ischemic stroke, acute intermediate high-risk pulmonary embolism, and radiological evidence of chronic popliteal vein thrombosis with an incidental finding of a complex atrial septal defect (ASD) with a left-to-right shunt.

## Case presentation

An 18-year-old female presented to the emergency department (ED) of Sultan Qaboos University Hospital (SQUH) with a one-hour history of right-side body weakness, facial asymmetry, and altered mental status after an episode of vomiting. There was no history of fever, respiratory symptoms, diarrheal illness, trauma, immobilization, surgical intervention, skin rash, or recent travel. Her medical background was remarkable for PCOS for three years, and she took norethisterone to control menorrhagia, which was ceased two months before this presentation for no obvious medical reason. Her family history is remarkable for a sudden unexplained cardiac death of her paternal uncle at the age of 40. She was vaccinated with two BNT162b2 mRNA COVID-19 vaccine doses, and her last dose was six months before the presentation.

On presentation to ED, her vitals were as follows: Glasgow Coma Scale (GCS) of 7/15, temperature of 36.8°C, blood pressure of 110/70 mmHg, respiratory rate of 20 per minute, pulse rate of 123 per minute, and oxygen saturation fluctuation between 70% and 98% in ambient air. She was obese (body mass index (BMI) of 30 kg/m^2^), with significant hirsutism. She had facial asymmetry; her pupils were equal and sluggish in response to light. Her plantar reflex response was equivocal bilaterally. Cardiovascular examination showed loud P2, but there was no murmur. There was no lower extremity swelling or edema. Other systemic examinations were unremarkable. Due to the patient’s critical condition and depressed conscious level, rapid sequence intubation was carried out.

**Figure 1:**
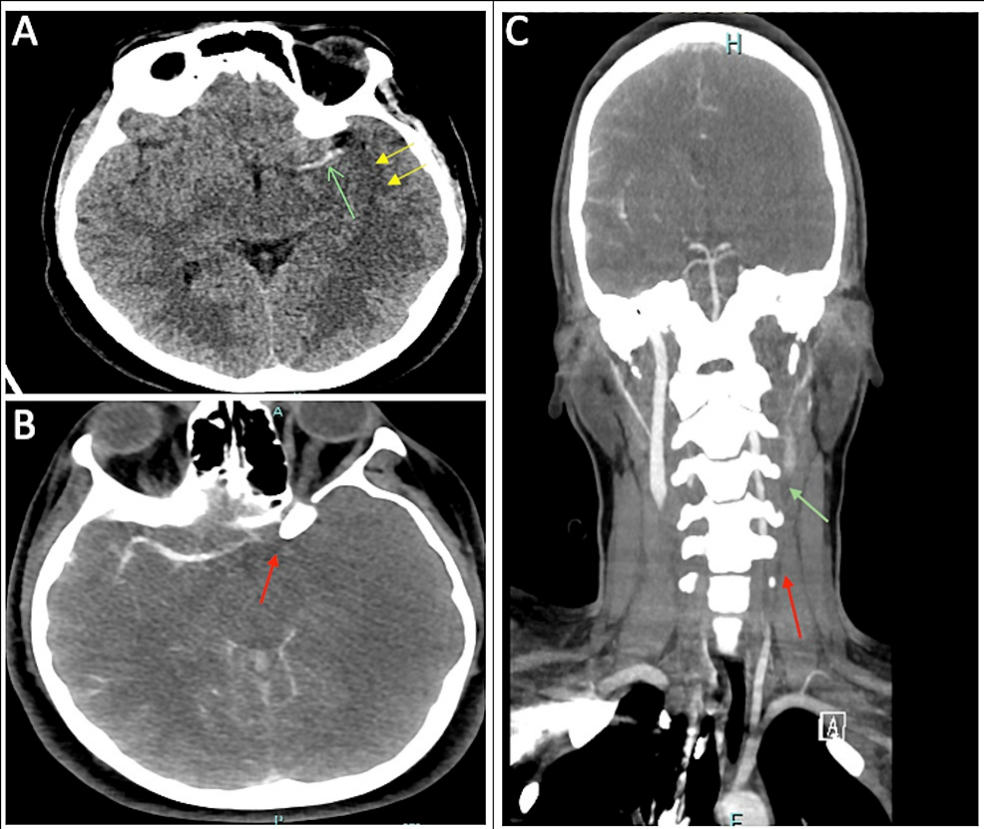
A: Hyperacute left MCA (green arrow) with loss of gray-white differentiation at insular and subinsular region (yellow arrows) suggestive of developing ischemic infarct in the territory of the left MCA. B: Filling defect involving the left MCA from segment M1 onward (red arrow ). C: Thrombosis of the left ICA just proximal to the bifurcation extending to the communicating segment (red arrow). MCA: middle cerebral artery, ICA: internal carotid artery

Computed tomography (CT) scan of the brain and cerebral angiography showed a filling defect involving the left middle cerebral artery (MCA) from segment M1 onward and loss of gray-white matter differentiation suggestive of diffuse brain edema and significant thrombosis of the left internal carotid artery (ICA) just proximal to the bifurcation (Figure 1A-1C).

In addition, 12-lead electrocardiography (ECG) showed sinus tachycardia with an S1Q3T3 pattern. Laboratory findings upon admission are presented in Table 1.

**Table 1:** Summary of laboratory test results on admission. Hb: hemoglobin, CRP: C-reactive protein, eGFR: estimated glomerular filtration rate

Test	Result	Normal range
Hematology		
Hb (g/L)	8	11.5-15.5
Hematocrit (L/L)	0.289	0.350-0.450
Platelet count (10^9^/L)	519	150-450
White cell count (10^9^/L)	27.4	2.2-10
Neutrophils (10^9^/L)	24.5	1-5
Biochemistry		
CRP (mg/L)	3	0-5
eGFR (mL/minute/1.73 m^2^)	>90	>90
Venous pH	7.232	7.35-7.45
PCO2 (mmHg)	49.2	36-48
HCO3 (mmol/L)	19	21.8-26.9
Lactate (mmol/L)	3.7	0.5-1.6
Anion gap	18	5-13
Electrolytes		
Potassium (mmol/L)	4.1	3.5-5.1
Sodium (mmol/L)	140	135-145
Calcium albumin adjusted (mmol/L)	2.3	2.15-2.55
Phosphate (mmol/L)	1.23	0.81-1.45

**Figure 2:**
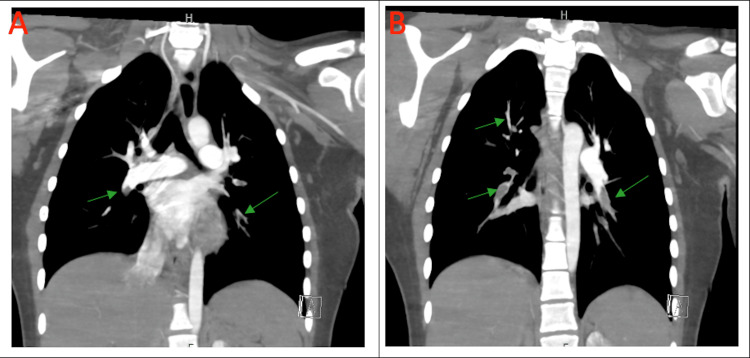
A and B: Bilateral lower lobe, right upper lobe, and left lingula segmental and sub-segmental filling defect (green arrows).

CT of the chest, abdomen, pelvis, and lower extremity revealed PE of segmental and subsegmental branches of bilateral lower, middle, and right upper lobe arteries with evidence of right heart strain and dilatation in the right popliteal vein with multiple collaterals suggestive of chronic thrombosis (Figure 2A and Figure 2B).

**Figure 3:**
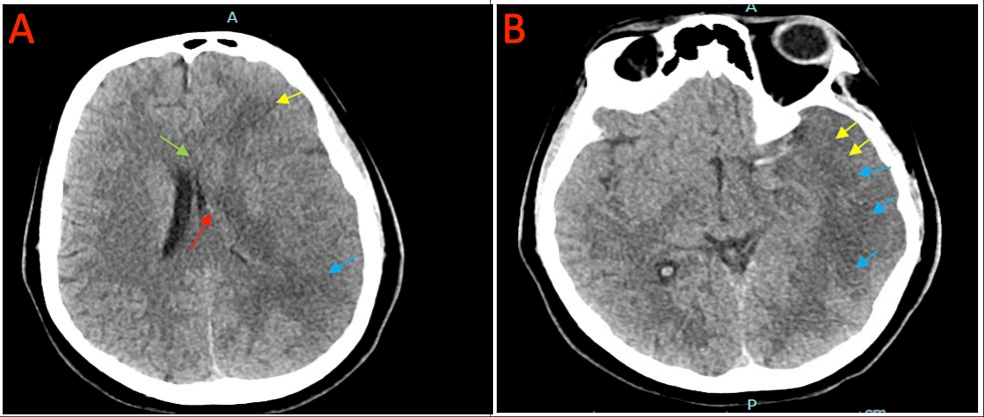
A and B: Loss of gray-white matter differentiation with sulcal effacement over the left MCA territory (yellow arrows) suggestive of acute infarct with diffuse brain edema. It shows an interval increase in the extension of the hypo-attenuation to involve the left occipital, left temporal, and left parietal lobe (blue arrows). There is an interval development of 5 mm midline shift to the right side (green arrow). There is a mass effect noted on the left lateral ventricle (red arrow). MCA: middle cerebral artery

An immediate multidisciplinary discussion involving the intensive care unit (ICU), neurology, pulmonology, hematology, and internal medicine units concluded that systemic thrombolysis, catheter-directed thrombectomy, and full anticoagulation carry a high risk of bleeding due to the massive brain ischemia. Due to the progression of brain edema (Figure 3A and Figure 3B), the patient was given mannitol and then underwent an urgent decompression craniotomy and inferior vena cava (IVC) filter insertion to prevent proximal embolization from lower limbs.

The evaluation for her underlying thrombosis burden was carried out and showed a high factor VIII (FVIII) level at 360%, borderline positive IgM for both beta-2-glycoprotein 1 (β2GP1) antibody and anticardiolipin (aCL) antibody, but negative finding for lupus anticoagulant, factor V Leiden (FVL), and prothrombin II G20220A mutation (Table 2).

**Table 2:** Summary of autoimmune and thrombosis workup. FV: factor V, LA: lupus anticoagulant, dRVVT: dilute Russell’s viper venom time, APTT: activated partial thromboplastin time

Test	Result	Normal range
Anti-deamidated gliadin antibody (IgA) (U/mL)	15	0-25
Anti-deamidated gliadin antibody (IgG) (U/mL)	2	0-25
Anti-transglutaminase antibody (IgA) (U/mL)	2	0-20
Anti-transglutaminase antibody (IgG) (U/mL)	<1	0-1
Antineutrophil cytoplasmic antibody	Negative	-
Antinuclear antibodies	Negative	-
Extractable nuclear antigens	Negative	-
Autoimmune antiphospholipid syndrome		
Anti-beta-2-glycoprotein 1 (IgG)	1	0-7
Anti-beta-2-glycoprotein 1 (IgM) (day 1)	30	0-7
Anti-beta-2-glycoprotein 1 (IgM) (week 16)	16	0-7
Anticardiolipin antibody (IgG)	4	0-10
Anticardiolipin antibody (IgM) (day 1)	17	0-10
Anticardiolipin antibody (IgM) (week 12)	12	0-10
Thrombophilia workup		
Antithrombin antigenic (u/mL)	0.789	0.800-1.400
Antithrombin function (IIa inhibition 20 seconds) (u/mL)	0.744	0.880-1.220
Antithrombin function (Xa inhibition) (u/mL)	0.645	0.890-1.280
Protein C functional chromogenic (u/mL)	0.530	0.720-1.540
Protein C antigenic (u/mL)	0.521	0.700-1.400
Protein S functional (u/mL)	0.744	0.520-1.180
Free protein S antigenic (u/mL)	0.718	0.601-1.136
ProC Global/FV	0.8	0.86-1.10
Factor VIII chromogenic assay (u/mL)	3.597	0.580-1.880
Lupus anticoagulant		
LA screen (dRVVT) (seconds)	58.3	31-46.2
LA screen mix (dRVVT) (seconds)	45.1	36-44.5
LA screen mix ratio (dRVVT)	1.2	1-1.1
LA confirm (dRVVT) (seconds)	52.1	28.1-36
LA screen (APTT-LA sensitive) (seconds)	47.2	31.1-49.8
LA screen ratio (dRVVT)	1.5	0.9-1.1
LA confirm ratio (dRVVT)	1.5	0.9-1.1

Furthermore, a concern of paradoxical embolization phenomenon from the heart was raised, given the involvement of both arterial and venous systems with thrombosis phenomenon. Therefore, transesophageal echocardiography (TEE) was done and showed inferior sinus venosus atrial septal defect (ASD), which is not amenable for endovascular repair; rather, a cardiothoracic intervention was advised after recovery from the acute illness.

Non-contrast follow-up brain CT was done on day 4 and showed no evidence of hemorrhagic transformation. Enoxaparin was introduced at a dose of 0.5 mg/kg twice daily and escalated to 1 mg /kg twice daily over a few days. Unfortunately, the patient had a seizure episode; therefore, levetiracetam 1,000 mg twice daily was initiated. Her ICU stay was complicated by ventilator-acquired pneumonia, resulting in septic shock that required prolonged courses of multiple broad-spectrum intravenous antibiotics.

She could not tolerate weaning off ventilation, for which a tracheostomy was performed two weeks after intubation. After 21 days of ICU admission, she was successfully shifted to a high-dependency room with extensive physiotherapy. A week later, she was shifted to a regular medical bed and decannulated from a tracheostomy tube, and enoxaparin was shifted to oral rivaroxaban 20 mg once daily as per the thrombosis specialist’s recommendation.

The patient continued to improve with physiotherapy, and toward the discharge, she was alert and responding well to commands and could mobilize with assistance. Finally, after 62 days, she was clinically and hemodynamically stable to be discharged home. Shared follow-up appointments were arranged with relevant specialities.

## Discussion

This case described a complex etiology and treatment approach for an 18-year-old female known case of untreated PCOS who presented to the hospital a few months after receiving the COVID-19 vaccine with extensive arterial and venous thrombosis that required prolonged ICU admission. Later during investigations, she was found to have ASD with a left-to-right shunt. In this case, the approach of differential diagnosis was around the etiologies that best fit the cause of both venous and arterial thrombosis and the consequences that lead to the stroke as paradoxical embolism. Workup for thrombophilia showed elevated factor VIII level, which can be associated with venous and arterial thrombosis, including stroke, and linked to increased risk of recurrence; however, during acute thrombosis events, the acute phase reaction may cause such elevation [16]. The workup for antiphospholipid syndrome (APS) revealed negative lupus anticoagulant with weak positive IgM only for anticardiolipin (aCL) antibodies and anti-beta-2-glycoproteins 1 (β2GP1) antibodies; both are less than 40 unit, which is clinically not significant for APS and does not fulfill all laboratory criteria. This mild elevation can be transient during acute thrombosis [17]. The follow-up test after 12 weeks showed down-trending titers of both IgM for anticardiolipin (aCL) antibodies and anti-beta-2-glycoproteins I (β2GP1) antibodies, which are not supporting the diagnosis of APS. Because of the absence of evidence of malignancy, autoimmune disorders, and inherited coagulopathies, we presumed that her untreated PCOS is the main predisposing factor leading to VTE. At the same time, the paradoxical embolism phenomenon due to ASD could explain the stroke.

PCOS could explain the patient presentation, as the significant association with stroke and other cardiometabolic diseases are well described in several meta-analyses from literature, especially with increased BMI [7,18]. Additionally, combined oral contraceptives (COCs) containing estrogen are well known to increase the risk of VTE and ischemic stroke. Furthermore, its risk is high in the first year of initiation, particularly in the sustained period of use [17,19], via the changes in hemostatic parameters caused by desogestrel-containing oral contraceptives [20]. However, the patient used to be on progestin-only pills (norethisterone) for menorrhagia, which was stopped two months before her presentation. On the other hand, there have been an increasing number of reports of COVID-19 vaccine-related thrombosis and a few reports of stroke, which were likely to occur a few days to a maximum of 28 days after the first dose of the vaccine, mainly in middle-aged women [8-10,12,13,15]. Vaccine-induced immune thrombotic thrombocytopenia (VITT) was highly linked to the ChAdOx1 nCoV-19 vaccine only [15]. However, the patient presented with a slightly higher than normal platelet count, which is inconsistent with VITT; hence, further testing was not pursued.

The paradoxical embolism phenomenon was a suggested mechanism to explain the stroke in our patient due to her complex inferior sinus venosus ASD. Although her ASD was clear left-to-right shunt, right ventricle strain secondary to the primary pulmonary embolism could cause transient elevation of the right heart pressure and right-to-left cardiac embolization [21].

A previous study demonstrated that venous thromboembolism (VTE) accounts for 0.20% of PCOS patients, and out of that small percentage, only 19.4% had both DVT and PE, which represents 0.038% of the total PCOS cases [22]. Moreover, PCOS is linked with a significantly increased risk of developing ischemic stroke [16,23]. However, combined DVT, PE, and stroke was not reported in a patient with PCOS previously, up to our knowledge.

Overall, the chain of events was proposed to start with chronic DVT, likely precipitated by PCOS and obesity. Then, she developed pulmonary embolism that triggers right ventricle strain and transient shunt reversal through the congenital defect (ASD), leading to a presumed paradoxical embolism phenomenon and a massive ischemic stroke.

## Conclusions

Polycystic ovarian syndrome can be associated with extensive venous and arterial thrombosis. We presented a case of a patient who had a stormy course of extensive venous and arterial thromboses. The decision of anticoagulation in the presence of acute ischemic stroke is complex and requires a multidisciplinary team approach to ensure a high standard of patient-centered care that leads to a good outcome in our case.
